# Colchicine Suppresses Adipogenic Differentiation of Mesenchymal Stem Cells: Implications for Bone Adiposity Control

**DOI:** 10.3390/pharmaceutics18010119

**Published:** 2026-01-16

**Authors:** Miriam López-Fagúndez, María Piñeiro-Ramil, Andrés Pazos-Pérez, María Guillán-Fresco, Verónica López, Djedjiga Ait Eldjoudi, Susana Belén Bravo, Alberto Jorge-Mora, Ana Alonso-Pérez, Rodolfo Gómez

**Affiliations:** Musculoskeletal Pathology Group, Health Research Institute of Santiago de Compostela (IDIS), Santiago University Clinical Hospital, SERGAS, 15706 Santiago de Compostela, Spain; miriam.lopez.fagundez@sergas.es (M.L.-F.); maria.ramil@idisantiago.es (M.P.-R.); andres.pazos.perez@sergas.es (A.P.-P.); maria.guillan.fresco@sergas.es (M.G.-F.); veronica.lopez.lopez1@sergas.es (V.L.); djedjiga.ait.eldjoudi@sergas.es (D.A.E.); susana.belen.bravo.lopez@sergas.es (S.B.B.); alberto.agustin.jorge.mora@sergas.es (A.J.-M.)

**Keywords:** adipogenesis, osteoblastogenesis, cytoskeleton, microtubule destabilisation, bone marrow adipocytes

## Abstract

**Background**: Gout is an inflammatory arthritis associated with increased bone anabolism and a higher risk of ectopic bone formation. Colchicine, used to prevent and treat acute gouty flares, inhibits microtubule polymerization and has been described to promote osteoblastogenesis. In bone disorders such as osteoporosis, disruption of the osteoblast–adipocyte balance contributes to pathology, yet no therapies directly target bone marrow adiposity. Thus, we decided to investigate the impact of colchicine on the osteoblast-adipocyte balance. **Methods**: C3H10T1/2 mesenchymal stem cells were differentiated to both cell fates in the presence or absence of colchicine. Differentiation was assessed by studying differentiation phenotypes as well as adipocytic and osteoblastic marker genes. Disrupting microtubule homeostasis through stathmin (STMN1) silencing was employed to mimic colchicine effects on differentiation. Proteomic analysis was performed to gain further insight into colchicine’s effects on adipogenesis. **Results**: Colchicine promoted transcriptional changes consistent with osteoblastogenic commitment and inhibited adipogenesis, as evidenced by reduced intracellular lipid accumulation and downregulation of adipogenic marker genes. These effects were observed following both continuous and transient exposure (median fold change across adipogenic markers 0.41 and 0.59, respectively). Consistent with colchicine-induced microtubule destabilisation, microtubule disruption by STMN1 silencing also suppressed adipogenic differentiation (median fold change = 0.66), suggesting that colchicine’s anti-adipogenic effect may be due to its impact on the cytoskeleton. **Conclusions**: These findings indicate that colchicine can suppress adipogenic differentiation while favouring osteoblast commitment in mesenchymal stem cells. Although further validation in relevant preclinical models is required, its efficacy following transient exposure supports the exploration of site-specific strategies that limit systemic toxicity.

## 1. Introduction

The rising prevalence of musculoskeletal disorders is an increasing concern for the well-being of the ageing population. Gout is the most common form of inflammatory arthritis, with a prevalence of 1–4% in the general population and up to 11–13% in people over 80 years of age [1]. Gout patients show high levels of uric acid, which leads to the formation of crystals in the joints, causing inflammation and pain. Radiological features of gout include tophi deposition, bone erosion, and new bone formation, with sclerosis and spurs being the most common lesions associated with this disease [2]. In regard to this, heterotopic ossification is a postoperative complication of arthroscopic surgery, with an incidence as high as 46% [3], but rising to 100% in gout patients [4]. Consistent with this elevated bone anabolism, higher uric acid levels in gout patients have been associated with a reduced risk of osteopenia [5].

Colchicine, a drug derived from the bulb-like corms of the Colchicum autumnale plant, is used to prevent or treat acute gouty flares and familial Mediterranean fever [6]. However, above the therapeutic range, colchicine can also induce toxic effects, including myopathy, neuropathy, and cardiovascular events [7]. Colchicine binds to tubulin and inhibits microtubule polymerization, which plays a critical role in cell structure, intracellular transport, and cell fate commitment [8], thus blocking the cell cycle at the G2/M phase and triggering apoptosis [9]. In addition, colchicine has been reported to modulate autophagy, although conflicting evidence exists as to whether it promotes autophagy or impairs autophagic flux [10,11,12]. Inhibition of microtubule assembly has also been described to increase bone morphogenetic protein 2 (BMP-2) expression and subsequent bone formation [13]. Interestingly, colchicine has also been demonstrated to promote osteoblastogenesis, inducing osteopontin (SPP1) expression and increasing alkaline phosphatase activity [14,15]. Most importantly, colchicine injection is enough to trigger ectopic bone formation in vivo [16,17]. In line with this, inhibition of stathmin (STMN1), a regulator of microtubule polymerization, also results in cell cycle arrest and affects osteoblastogenic differentiation [18].

Osteoblastogenesis and adipogenesis are antithetical and mutually controlled processes [19]. Thus, the promotion of osteoblastogenesis may inhibit adipogenesis and vice versa. In bone diseases such as osteoporosis and in conditions like obesity and ageing, disruption of the balance between osteoblastogenesis and adipogenesis plays a key role in the associated bone alterations. Specifically, increased bone marrow adiposity is a common feature of several bone disorders and has been associated with poor bone quality and a higher risk of fractures [20]. Despite current therapies aimed at inhibiting bone resorption (e.g., bisphosphonates, denosumab) or stimulating bone formation (e.g., teriparatide, romosozumab), no treatment specifically targets bone marrow adiposity. In this context, the effects of colchicine on adipogenesis remain unexplored.

In this study, we investigated whether colchicine treatment and microtubule disruption could control the osteoblast-adipocyte balance. We demonstrated that therapeutic concentrations of colchicine [21] alter the osteoblast-adipocyte balance, inhibiting adipogenesis while increasing the expression of osteoblast marker genes. In addition, microtubule disruption caused by STMN1 silencing recapitulated this inhibitory effect on adipogenesis. Based on these results, we suggest that colchicine warrants further investigation as a modulator of the osteoblast-adipocyte balance in bone pathologies related to an increased bone marrow adiposity, such as osteoporosis.

## 2. Materials and Methods

### 2.1. Reagents

The murine mesenchymal stem cell line C3H10T1/2 was donated by Dr. Pardo’s group (IDIS). Dulbecco’s Modified Eagle’s Medium High Glucose (DMEM-HG, Sigma-Aldrich, Cat#D5796), Dulbecco’s Modified Eagle’s Medium supplemented with Ham’s F-12 (DMEM F12, Sigma-Aldrich, Cat#D8437), Foetal Bovine Serum (FBS, Sigma-Aldrich, Cat#F7524), antibiotics Penicillin-Streptomycin (Sigma-Aldrich, Cat#P0781), L-Glutamine (Sigma-Aldrich, Cat#G7513), Trypsin (Sigma-Aldrich, Cat#T4049), MTT reagent (Sigma-Aldrich, Cat#M2128), chloric acid (Sigma-Aldrich, Cat#C2423), TRI-Reagent (Sigma-Aldrich, Cat#T9424), rosiglitazone (Sigma-Aldrich, Cat#R2408), dexamethasone (Sigma-Aldrich, Cat#D4902), indomethacin (Sigma-Aldrich, Cat#I7378), 3-isobutyl-1-methylxanthine (IBMX, Sigma-Aldrich, Cat#I5879), β-glycerol phosphate (Sigma-Aldrich, Cat#G9422), ascorbic acid-2-phosphate (Sigma-Aldrich, Cat#A8960), Alizarin Red S (Sigma-Aldrich, Cat#A5533), Oil Red O (Sigma-Aldrich, Cat#O0625), and colchicine (Sigma-Aldrich, Cat#C9754, purity ≥ 95%) were purchased from Sigma-Aldrich (St. Louis, MO, USA). Insulin-like growth factor-1 (IGF-1, PeproTech Inc., Cat#250-19, Cranbury, NJ, USA) was used at 20 nM.

The E.Z.N.A. Total RNA Kit I was bought from Omega BIO-TEK (Norcross, GA, USA; Cat# R6834). RNase-Free Dnase I was purchased from Lucigen (Middleton, WI, USA; Cat# D9905K). The High-Capacity RNA-To-cDNA Kit was obtained from Thermo Fisher Scientific (Thermo Fisher Scientific, Waltham, MA, USA; Cat# 4387406). MasterMix PCR was purchased from Bio Rad (BioRad Laboratories, Hercules, CA, USA; Cat# 172-5121). All primers were obtained from Sigma-Aldrich (Supplementary Table S1).

The STMN1 silencing reagents were obtained from a specific TriFECTa DsiRNA Kit (Integrated DNA Technologies, Coralville, IO, USA), and the siLentFect^TM^ Lipid Reagent for RNAi was purchased from BioRad (BioRad Laboratories; Cat# 170-3360). Opti-MEM (Cat# 31985-062), *Cell Mask*^TM^ *Green Actin Tracking Stain* kit (Cat# R37110), Tubulin Tracker TM Deep Red kit (Cat# T34076), and NucBlue™ Live ReadyProbes™ kit (Cat# R37605) were obtained from Gibco (Thermo Fisher Scientific).

### 2.2. Cell Culture and Differentiation

C3H10T1/2 cells were grown in DMEM-HG supplemented with 10% FBS, 1% penicillin-streptomycin, and 2% L-glutamine (DMEM10%FBS). For their differentiation, 10^4^ cells were seeded per well in a 24-well plate (Thermo Fisher Scientific; Cat# 142475). Differentiation media were added 6 h after seeding, except for STMN1 silencing experiments, in which differentiation media were added the following day.

Adipogenic differentiation medium (AD) was composed of DMEM10%FBS supplemented with 2 μM rosiglitazone, 20 nM IGF1, 1 μM dexamethasone, 60 μM indomethacin, 0.5 mM IBMX, and 10 μg/mL insulin. Adipogenic maintenance medium (ADm) consisted of DMEM10%FBS supplemented with 10 μg/mL insulin. Culture medium was refreshed every 2–3 days until achieving a 7-day adipogenic differentiation. AD medium was added on days 0 and 2 of differentiation, while ADm medium was employed on day 4 of differentiation.

Osteoblastogenic differentiation medium (OB) consisted of DMEM10%FBS supplemented with 10 nM dexamethasone, 5 mM β-glycerol phosphate, and 50 μg/mL ascorbic acid-2-phosphate. Culture medium was refreshed every 2–3 days until achieving a 7-day osteoblastogenic differentiation.

To study the impact of colchicine on differentiation, C3H10T1/2 cells were exposed to colchicine (2.5 µM and 25 nM) throughout adipogenic and osteoblastogenic differentiation. The medium containing colchicine was renewed every 2–3 days, following the differentiation protocol. After that, samples were processed for real-time PCR and proteomic analysis.

To examine the effect of a single pulse of colchicine (transient exposure experiments), C3H10T1/2 cells were exposed to colchicine (25 nM) during the first two days of adipogenic differentiation. Subsequently, for the ensuing 5 days of the experiment, the drug was removed, and C3H10T1/2 cells were exclusively subjected to adipogenic differentiation media.

### 2.3. Cell Viability

Cell viability was tested using the MTT reagent (3[4,5dimthylthiazol-2-yl]-2-5diphenyltetrazolium bromide) [22]. Cells (8 × 10^3^ per well) were seeded in 96-well plates (Thermo Fisher Scientific; Cat# 130188) and allowed to attach for 6 h, after which the culture medium was replaced with serum-free medium. The assay was performed following the manufacturer’s instructions. Briefly, on the day after seeding, cells were treated with colchicine in either DMEM10%FBS or in serum-free medium for 48 h at 37 °C. Following treatment, cells were incubated with 10 µL of MTT solution (5 mg/mL) for 4 h at 37 °C. After dissolution of the formazan crystals, absorbance was measured at 550 nm with a Multiskan SkyHigh microplate spectrophotometer (Thermo Fisher Scientific).

### 2.4. Cell Staining

The histochemical techniques Alizarin Red and Oil Red O were performed on fixed cells as previously described [23] and photographed using a Leica DMi1 microscope (Wetzlar, Germany). Oil Red O staining was quantified in Fiji (ImageJ version 1.54p) as the percentage of stained area (area fraction) within the field of view using a fixed thresholding workflow applied identically to all images.

To study the differences in the cytoskeleton induced by colchicine stimulation, C3H10T1/2 cells were seeded on 15 nm diameter coverslips (Thermo Fisher Scientific) on 24-well plates. Six hours after seeding, cells were treated with two colchicine doses (2.5 μM and 25 nM) for 2 and 48 h, respectively. After this time, C3H10T1/2 cells were stained. *Cell Mask*^TM^ *Green Actin Tracking Stain* kit was used to stain actin. The *Tubulin Tracker*^TM^ *Deep Red* kit was used to stain tubulin. Finally, the *NucBlue™ Live ReadyProbes™* kit was applied to stain the nuclei. All these kits were used according to the manufacturers’ instructions. Cells were observed and photographed using a Leica TCS SP8 confocal microscope (Wetzlar, Germany).

### 2.5. STMN1 Silencing

C3H10T1/2 cells were seeded at a density of 10^4^ cells per well on 24-well plates. The following day, the culture medium was changed to DMEM F12 containing 2% FBS. After 1 h incubation, STMN1 silencing was performed using the *TriFECTa DsiRNA Kit* (15 nM) and *the siLentFect™ Lipid Reagent for RNAi* in Opti-MEM. A negative control DsiRNA was included to confirm that the transfection process did not affect normal cell function. After 6 h incubation, the medium was discarded, and the cells were subjected to adipogenic and osteoblastic differentiation as previously described. Cells were lysed for RNA extraction after 7 days of differentiation.

### 2.6. RNA Extraction and Quantitative Real-Time PCR

Once the experiments were concluded, cells were lysed, and RNA was extracted using TRI-Reagent and E.Z.N.A. Total RNA Kit I, according to the manufacturer’s instructions. Afterwards, RNA was purified using the RNase-Free DNase I. The High-Capacity RNA-To-cDNA Kit was used to retrotranscribe 500 ng of RNA.

The expression of STMN1, osteopontin (SPP1), leukaemia inhibitory factor (LIF), transmembrane glycoprotein NMB (GPNMB), runt-related transcription factor 2 (RUNX2), peroxisome proliferator-activated receptor gamma (PPARY), perilipin 2 (PLIN2), adiponectin (ADIPOQ), and fatty acid-binding protein 4 (FABP4) was measured by real-time PCR (RT-qPCR) using the MasterMix PCR in combination with specific KiCqStart™ primer pairs (Supplementary Table S1) in a Stratagene Mx 3005P Thermocycler (Stratagene, La Jolla, CA, USA). To analyse the amplification and melt curves, the MxPro software v4.10 (Stratagene) was used. RT-qPCR data relative quantitation was obtained through the ΔΔCt comparative method, and hypoxanthine phosphoribosylltransferase 1 (HPRT) was used as the housekeeping gene (Supplementary Figure S1).

### 2.7. Qualitative and Quantitative Proteomic Analysis

Protein extracts from the adipogenic differentiation protocol described in Section 2.2 (including a day 0 control, a day 7 adipogenesis control, and a day 7 adipogenesis sample treated with 25 nM colchicine) were subjected to proteomic analysis. Protein extracts were prepared by lysing cells in RIPA buffer (Millipore, Sigma-Aldrich) supplemented with a protease and phosphatase inhibitor cocktail (Thermo Fisher Scientific), followed by clarification of the lysates by centrifugation at 18,000× *g* for 20 min at 4 °C. Data-dependent acquisition (DDA) analysis was performed on a TripleTOF 6600 mass spectrometer (SCIEX, Redwood City, CA, USA). DDA runs were processed with ProteinPilot™ v5.0.1 (SCIEX) using the Paragon algorithm against the UniProt Mouse database. Only peptides with a confidence score above 99% (as obtained from Protein Pilot database search FDR < 1%) were included in the spectral library.

The resulting spectral library was used for Sequential Window Acquisition of All Theoretical Mass Spectra (SWATH-MS) analyses on the same instrument [24]. The SWATH–MS method is based on repeating a cycle consisting of the acquisition of 100 TOF MS/MS scans (400–1500 *m*/*z*, high-sensitivity mode, 50 ms acquisition time) of overlapping sequential precursor isolation windows of variable width (1 *m*/*z* overlap) covering the 400–1250 *m*/*z* mass range, with a previous TOF MS scan (400–1500 *m*/*z*, 50 ms acquisition time) for each cycle. The total cycle time was 6.3 s. The width of the 100 variable windows was optimised according to the ion density found in the DDA runs using the SWATH–MS variable window calculator worksheet from Sciex.

The targeted data extraction of the fragment ion chromatogram traces from the SWATH–MS runs was performed by PeakView (version 2.2) using the SWATH–MS Acquisition MicroApp (version 2.0) over the spectral library created from the DDA data. Up to ten peptides per protein and seven fragments per peptide were selected based on the signal intensity to obtain the peak areas, and any shared and modified peptides were excluded from processing.

Integrated peak areas (SWATH–MS areas) were directly exported to MarkerView software (Version 1.3) (Sciex, Redwood City, CA, USA) for a relative quantitative analysis. MarkerView uses processing algorithms that accurately identify chromatographic and spectral peaks directly from raw SWATH data. First, the integrated peak areas were normalised using MLR normalisation on the analysis performed, and an unsupervised multivariate statistical analysis using principal component analysis (PCA) was performed to compare the data across the samples using scaling. The dysregulated proteins were selected using *p* < 0.05 and |log2FC| ≥ 0.5 as a threshold. No additional multiple-testing correction was applied to the SWATH quantitative analysis. All the data generated were processed using R Studio software v2023.12. Venn diagrams were generated on Funrich software v3.1.3. A detailed description of protein identification and quantification by LC–MS/MS DDA and SWATH–MS analyses is provided as Supplementary Methods.

### 2.8. Statistical Analysis

All statistical analyses were performed using GraphPad Prism software v9.5.0 (GraphPad Software Inc., La Jolla, CA, USA). For experiments involving more than two experimental groups (e.g., MTT assays), one-way ANOVA followed by Bonferroni’s post hoc test was applied to correct for multiple comparisons. For predefined pairwise comparisons addressing specific hypotheses, Student’s *t*-test was used. Data are presented as mean ± standard error of the mean (SEM) for at least three independent experiments, and *p* < 0.05 was considered statistically significant. To summarise coordinated changes in adipogenic gene expression, log_2_-transformed fold changes for PPARγ, PLIN2, ADIPOQ, and FABP4 were averaged per gene across biological replicates, and the median across genes was calculated, and values were back-transformed (2^median) for reporting.

## 3. Results

### 3.1. Colchicine Alters the Osteoblast-Adipocyte Balance

**Figure 1:**
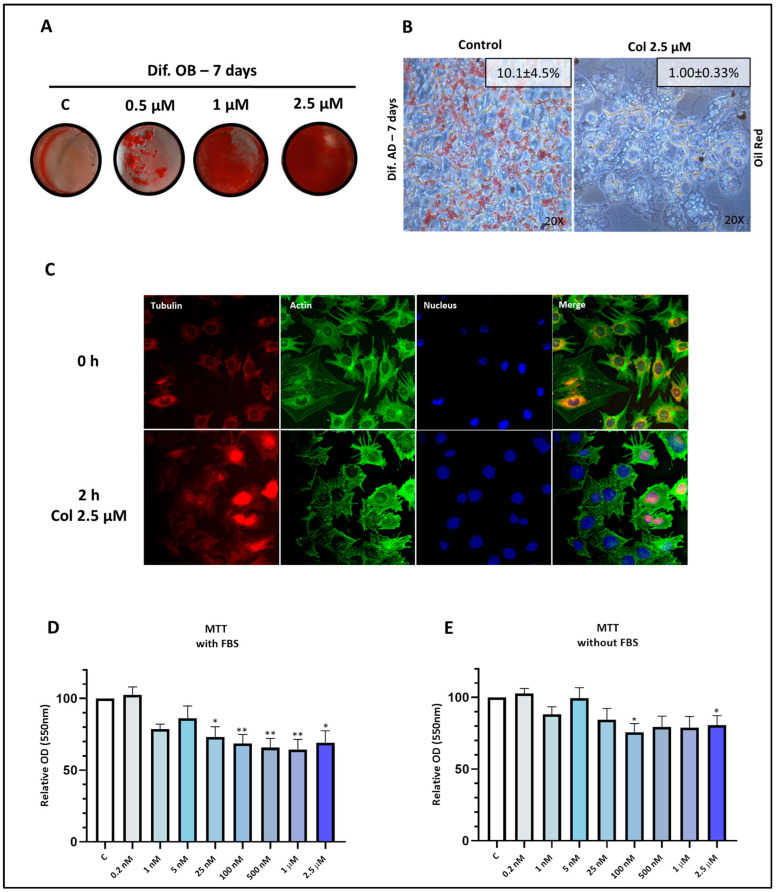
Colchicine treatment alters osteoblast-adipocyte balance. (**A**) C3H10T1/2 cells were subjected to differentiation in OB medium for 7 days with 2.5 µM colchicine and stained with Alizarin Red. (**B**) C3H10T1/2 cells were subjected to differentiation in AD medium for 7 days with 2.5 µM colchicine and stained with Oil Red O. Representative images are shown, together with quantification of the Oil Red O–positive area (% area, mean ± SEM). (**C**) The impact of colchicine stimulation on microtubule structure was assessed in C3H10T1/2 cells. Cells were stimulated with 2.5 µM colchicine for 2 h, and immunofluorescent images of tubulin, actin, and DAPI were merged using a confocal microscope system. (**D**) To evaluate the effect on cell proliferation, C3H10T1/2 cells were incubated with colchicine (in FBS-enriched medium) for 48 h, and an MTT assay was performed (n = 3). (**E**) C3H10T1/2 cells were incubated with colchicine (in FBS-depleted medium) for 48 h to assess their impact on cell viability through an MTT assay (n = 3). MTT: (3[4,5dimthylthiazol-2-yl]-2-5diphenyltetrazolium bromide). FBS: Foetal bovine serum. OD: Optical density. The data presented represents the fold change over control data and is expressed as the mean ± SEM, following at least three independent experiments (*p* value—GP style: *p* < 0.05 was indicated as *, *p* < 0.01 as **).

Several studies have suggested a potential correlation between gout, colchicine consumption, and increased bone density and bone anabolism [2,25]. In line with these findings, we investigated the effect of colchicine on the osteoblast-adipocyte balance. To assess this, C3H10T1/2 cells were differentiated for 7 days to osteoblasts and adipocytes in the presence of supra-physiological high doses of colchicine, frequently used in vitro in the literature (up to 2.5 µM). As expected, colchicine promoted mineralization during osteoblastogenic differentiation (Figure 1A). Interestingly, colchicine also impaired the formation of lipid droplets during adipogenic differentiation (Figure 1B), suggesting its potential as an anti-adipogenic agent. Colchicine-treated cells displayed Oil Red O–negative vacuole-like cytoplasmic structures, likely reflecting cellular stress at the high concentration used (Figure 1B).

### 3.2. Colchicine Alters the Cytoskeleton Structure

Since colchicine is known to inhibit microtubule polymerization [26] and alter actin polymerization dynamics [27], we examined the impact of 2.5 µM colchicine on the cytoskeleton by means of tubulin and actin staining. Notably, colchicine induced the sequestering of tubulin in the nucleus. In addition, the structure of actin stress fibres was disrupted after 2 h of colchicine stimulation (Figure 1C).

### 3.3. Therapeutic Concentrations of Colchicine Are Cytostatic but Not Cytotoxic

It is widely acknowledged that colchicine has pro-osteoblastic properties [28,29]. However, the concentrations described in the literature for these in vitro properties are not representative of clinical practice concentrations and are potentially associated with toxic effects. Since clinical practice serum concentrations range from 7 to 30 nM [21], we conducted an MTT assay to identify colchicine concentrations without cytotoxic effects in vitro. MTT assay with FBS supplementation (cell division allowed) revealed a cytostatic effect of colchicine at concentrations starting from 25 nM (Figure 1D). MTT assay without FBS supplementation (cell division suppressed) showed that concentrations below 100 nM did not induce a cytotoxic response (Figure 1E). We selected the concentration of 25 nM to perform further experiments because it was within a relevant experimental range close to reported therapeutic levels and had a cytostatic effect without a cytotoxic effect, which indicates its ability to alter the cytoskeleton and, therefore, potentially modify cell differentiation commitment.

### 3.4. A Therapeutic Concentration of Colchicine Increases the Expression of Osteogenic Markers and Inhibits Adipogenesis

**Figure 2:**
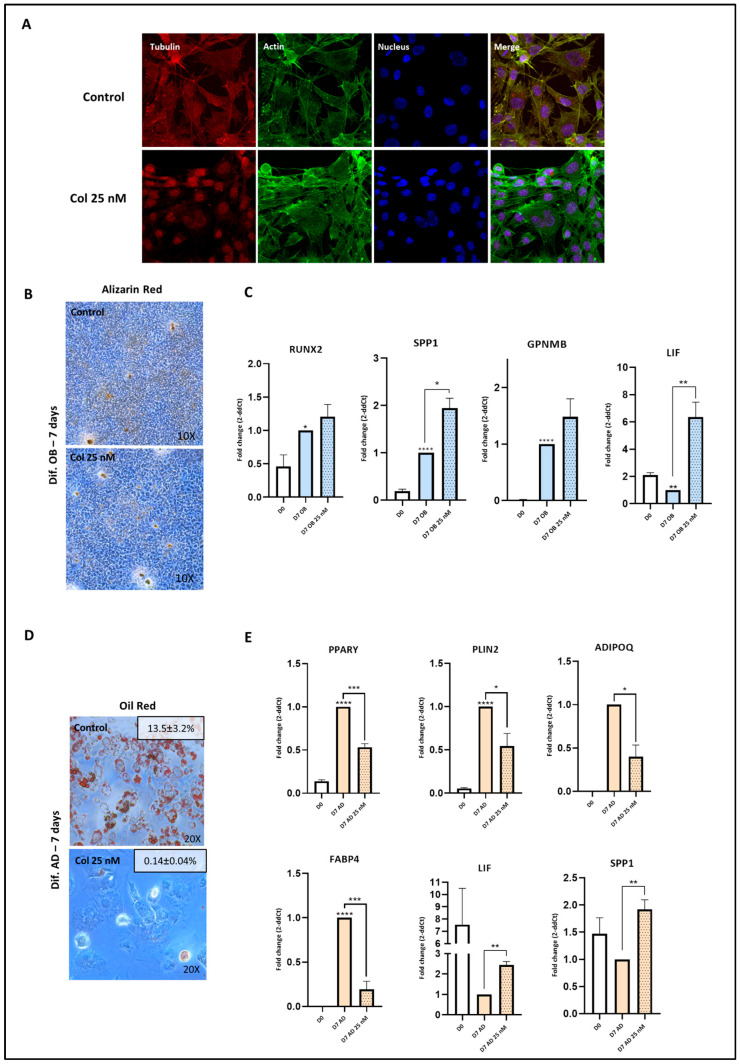
Therapeutic concentrations of colchicine increase the expression of osteoblast marker genes while inhibiting adipogenesis. (**A**) The impact of colchicine stimulation on microtubule structure was investigated. C3H10T1/2 cells were exposed to colchicine (25 nM) for 48 h, and subsequently, immunofluorescent images of tubulin, actin, and DAPI were merged using a confocal microscope system. (**B**) C3H10T1/2 cells were subjected to differentiation with OB medium with 25 nM colchicine for 7 days and stained with Alizarin Red. (**C**) Over a 7-day differentiation period with OB medium (n = 3), C3H10T1/2 cells were assessed for the expression of the osteoblast marker genes runt-related transcription factor 2 (RUNX2), osteopontin (SPP1), osteoactivin (GPNMB), and leukaemia inhibitory factor (LIF) using RT-qPCR. (**D**) C3H10T1/2 cells were differentiated with AD medium with 25 nM colchicine for 7 days and stained with Oil Red O. Representative images are shown, together with quantification of the Oil Red O–positive area (% area, mean ± SEM). (**E**) Over a 7-day differentiation period with AD medium (n = 3), C3H10T1/2 cells were evaluated for the expression of the genes peroxisome proliferator activated receptor gamma (PPARγ), perilipin 2 (PLIN2), adiponectin (ADIPOQ), fatty acid binding protein (FABP4), leukaemia inhibitory factor (LIF), and osteopontin (SPP1) using RT-qPCR. The presented data represent the fold change over control data for D7 differentiation and are expressed as the mean ± SEM, following at least three independent experiments (*p* value—GP style: *p* < 0.05 was indicated as *, *p* < 0.01 as **, *p* < 0.001 as ***, and *p* < 0.0001 as ****).

Next, we investigated whether the pro-osteoblastogenic and anti-adipogenic effects of colchicine were maintained at a clinical, non-toxic concentration (25 nM). Given that the cytoskeleton can regulate cell differentiation commitment [30,31], we explored the effect of this concentration of colchicine on cytoskeleton homeostasis. Tubulin staining revealed that colchicine treatment increased tubulin nuclear localization in C3H10T1/2 cells (Figure 2A). Regarding osteoblast differentiation, exposure to colchicine did not elicit any significant changes in C3H10T1/2 cells’ mineralization after 7 days of differentiation (Figure 2B). Osteoblastogenic differentiation noticeably upregulated the expression of osteoblast marker genes, namely SSP1, RUNX2, and GPNMB. Notably, colchicine stimulation further enhanced the expression of SPP1 and increased the expression of LIF (Figure 2C), a factor known to play a role in bone formation and adipogenesis inhibition [32,33]. Consistent with this, exposure to colchicine during adipogenic differentiation caused a striking reduction in the formation of lipid droplets (Figure 2D). This effect was accompanied by a strong inhibition of the expression of all the adipogenic marker genes studied, namely PPARγ, PLIN2, ADIPOQ, and FABP4 (median fold change = 0.41). Furthermore, colchicine exposure during adipogenic differentiation significantly increased the expression of LIF and SSP1 (Figure 2E).

### 3.5. STMN1 Silencing Does Not Modulate Osteoblastogenesis but Inhibits Adipogenesis

**Figure 3:**
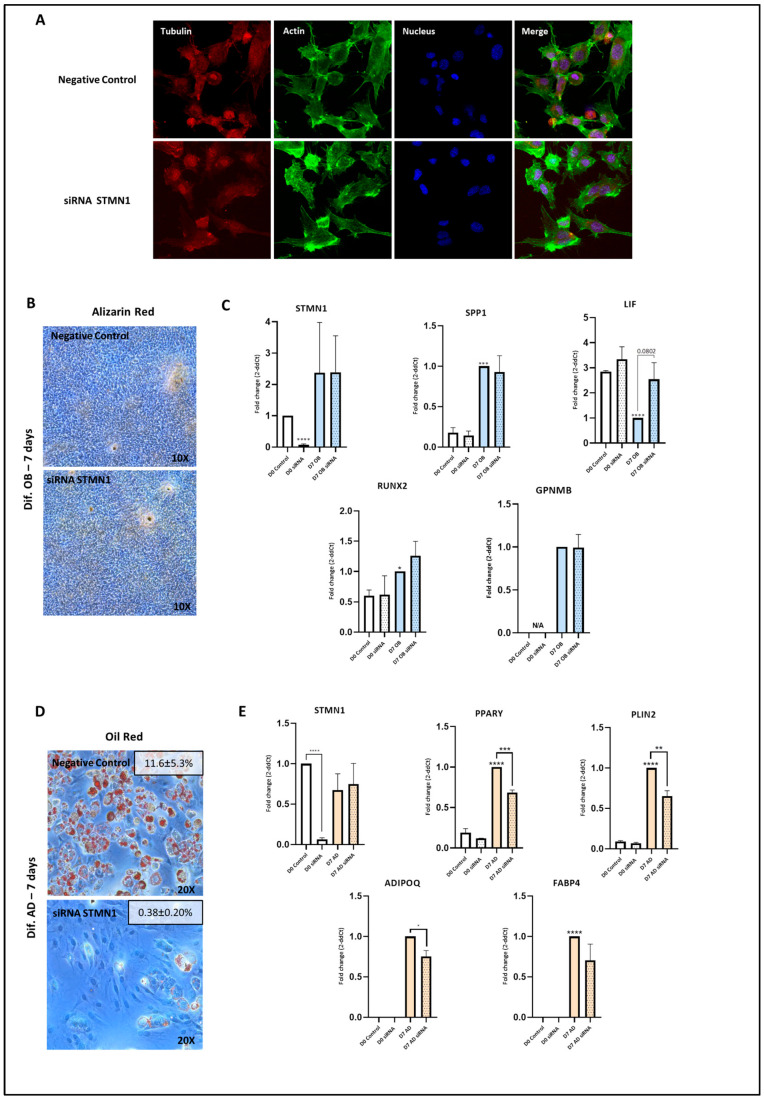
STMN1 silencing modifies osteoblast-adipocyte balance through microtubule structure. (**A**) The impact of STMN1 silencing on microtubule structure was examined. C3H10T1/2 cells were exposed to STMN1 silencing for 48 h, and subsequently, immunofluorescent images of tubulin, actin, and DAPI were merged using a confocal microscope system. (**B**) C3H10T1/2 cells were differentiated with OB medium for 7 days after STMN1 silencing. Cells were stained with Alizarin Red. (**C**) C3H10T1/2 cells were differentiated with OB medium for 7 days after STMN1 silencing (n = 3), and the expression of the genes stathmin 1 (STMN1), osteopontin (SPP1), leukaemia inhibitory factor (LIF), runt-related transcription factor 2 (RUNX2), and osteoactivin (GPNMB) was assessed by RT-qPCR. (**D**) C3H10T1/2 cells were differentiated with AD medium for 7 days after STMN1 silencing. Cells were stained with Oil Red O. Representative images are shown, together with quantification of the Oil Red O–positive area (% area, mean ± SEM). (**E**) C3H10T1/2 cells were differentiated with AD medium for 7 days after STMN1 silencing (n = 3), and the expression of genes stathmin 1 (STMN1), peroxisome proliferator-activated receptor gamma (PPARγ), perilipin 2 (PLIN2), adiponectin (ADIPOQ), and fatty acid binding protein (FABP4) was assessed by RT-qPCR. The presented data represent the fold change over control data for D7 differentiation and are expressed as the mean ± SEM, following at least three independent experiments (*p* value—GP style: *p* < 0.05 was indicated as *, *p* < 0.01 as **, *p* < 0.001 as ***, and *p* < 0.0001 as ****). N/A, not detected by RT-qPCR.

Considering the involvement of cytoskeleton dynamics in cell differentiation commitment [34] and the essential role of STMN1 in microtubule homeostasis [35], we aimed to determine whether microtubule metabolism disruption through STMN1 silencing could replicate the effects of colchicine during cell differentiation in C3H10T1/2 cells. Regarding the cytoskeleton, tubulin staining revealed that STMN1 silencing increased nuclear tubulin localization in a manner similar to colchicine treatment (Figure 3A).

Similarly to colchicine stimulation, STMN1 silencing did not induce a significant mineralization change in C3H10T1/2 cells differentiated into osteoblasts for 7 days (Figure 3B). In these cells, expression of osteoblast marker genes was not modulated by STMN1 silencing either. It was noteworthy that, even though STMN1 silencing was efficient, STMN1 expression was recovered during osteoblast differentiation (Figure 3C).

Similarly to the effect exhibited by colchicine, during adipogenic differentiation, STMN1 silencing promoted phenotypical differences, reducing the number of lipid droplets (Figure 3D). Consistent with this, the expression of the adipogenic marker genes PPARƳ, PLIN2, and ADIPOQ was also reduced by STMN1 silencing (median fold change = 0.66) (Figure 3E).

### 3.6. Transient Stimulation with Colchicine Exhibits Potent Anti-Adipogenic Effects

**Figure 4:**
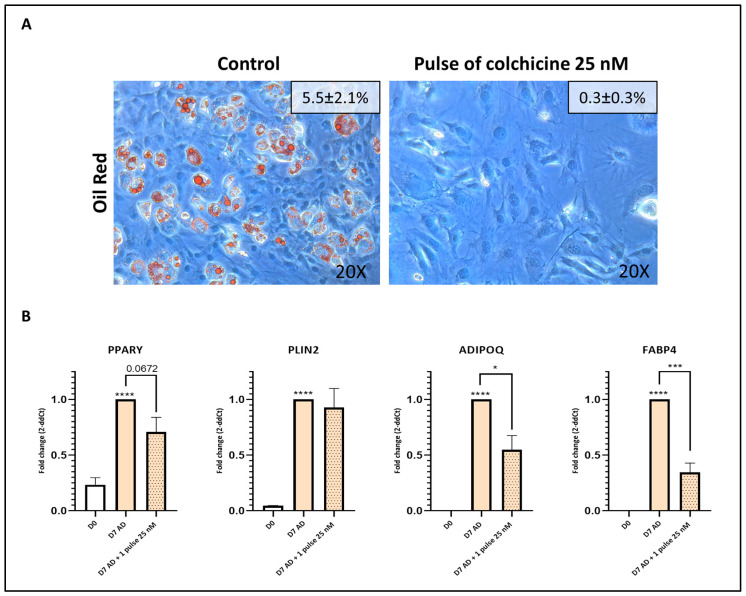
A transient pulse of colchicine inhibits adipogenesis. (**A**) C3H10T1/2 cells were differentiated with AD medium for 7 days after receiving a single pulse of colchicine during the first 48 h of differentiation (days 0–2), and Oil Red O staining was performed. Representative images are shown, together with quantification of the Oil Red O–positive area (% area, mean ± SEM). (**B**) C3H10T1/2 cells were differentiated with AD medium for 7 days following transient colchicine exposure (days 0–2) (n = 4), and the expression of the genes peroxisome proliferator-activated receptor gamma (PPARγ), perilipin 2 (PLIN2), adiponectin (ADIPOQ), and fatty acid binding protein (FABP4) was evaluated by RT-qPCR. The presented data represent the fold change over control data for D7 differentiation and are expressed as the mean ± SEM, following at least three independent experiments (*p* value—GP style: *p* < 0.05 was indicated as *, *p* < 0.001 as ***, and *p* < 0.0001 as ****).

To emulate STMN1 silencing, which lasts only 48–72 h, we treated C3H10T1/2 cells with a transient stimulation of colchicine during the first 48 h of adipogenic differentiation. This stimulation fully impaired the formation of lipid droplets (Figure 4A). Although colchicine treatment did not modify the expression of the adipogenic markers PPARƳ and PLIN2, it significantly decreased the expression of ADIPOQ and FABP4 (median fold change across adipogenic markers = 0.59) (Figure 4B).

### 3.7. Colchicine Alters the Expression of Proteins Associated with Microtubule Stability, Adipogenic Differentiation, and Autophagy

**Figure 5:**
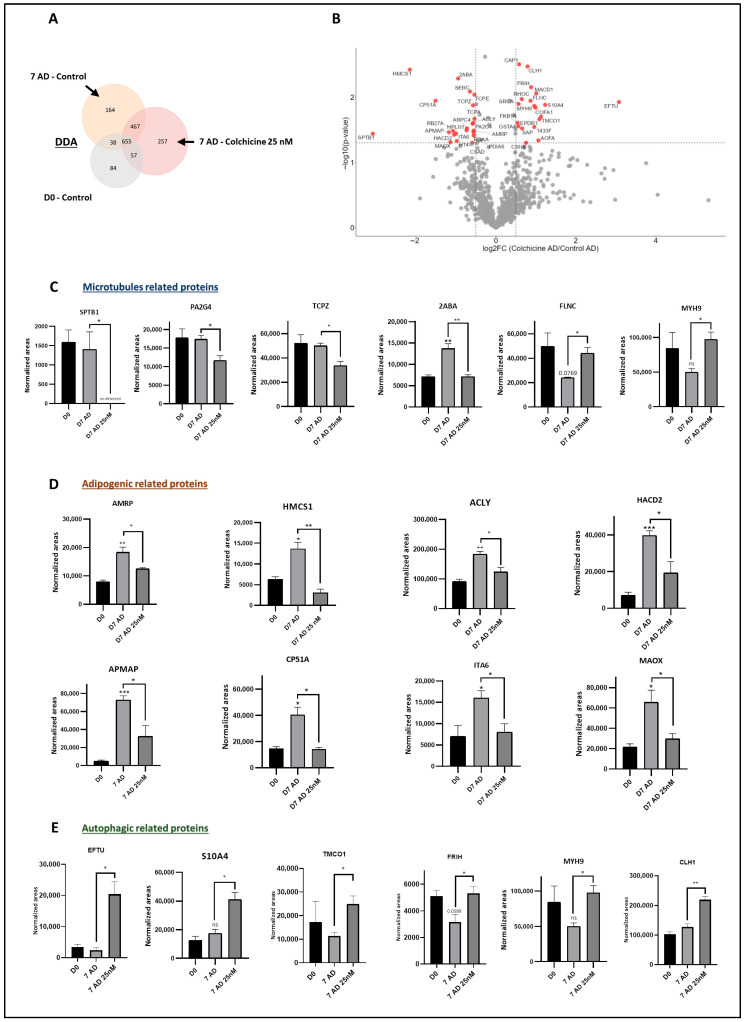
Colchicine stimulation inhibits the expression of proteins associated with adipogenesis. (**A**) Venn diagram illustrating colchicine effect over the proteins detected in qualitative analysis (DDA) (n = 3). (**B**) Quantitative data (SWATH) is represented in a volcano plot (log_10_ *p*-value vs. log_2_-fold change), illustrating protein expression changes induced by colchicine in an adipogenic environment (n = 3). (**C**) Assessment of microtubule-related protein expression levels after colchicine stimulation (SWATH) (n = 3): spectrin beta (STB1), proliferation-associated protein 2G4 (PA2G4), t-complex protein 1 subunit zeta (TCPZ), serine_threonine-protein phosphatase 2A (2ABA), filamin-C (FLNC), and myosin-9 (MYH9). (**D**) Analysis of adipogenic protein expression levels following colchicine stimulation (SWATH) (n = 3): alpha-2-macroglobulin receptor-associated protein (AMRP), hydroxymethylglutaryl-CoA synthase (HMCS1), ATP-citrate synthase (ACLY), very-long-chain (3R)-3-hydroxyacyl-CoA dehydratase 2 (HACD2), adipocyte plasma membrane-associated protein (APMAP), lanosterol 14-alpha demethylase (CP15A), integrin alpha-6 (ITA6), and NADP-dependent malic enzyme (MAOX). (**E**) Evaluation of autophagic-related protein expression levels following colchicine stimulation (SWATH) (n = 3): elongation factor Tu (EFTU), protein S100-A4 (S10A4), calcium load-activated calcium channel (TMCO1), ferritin heavy chain (FRIH), myosin-9 (MYH9), and clathrin heavy chain (CLH1). All data are derived from a minimum of three independent experiments and are presented as the Mean ± SEM (*p* value—GP style: *p* < 0.05 was indicated as *, *p* < 0.01 as **, *p* < 0.001 as ***). ns, not statistically significant compared to D0.

To achieve further insights into the inhibitory effect of colchicine on adipogenesis, we studied the proteomic changes elicited by this drug on C3H10T1/2 cells during adipogenic differentiation. Colchicine treatment disrupted the adipogenic process by altering the expression of numerous proteins regulated during adipocyte differentiation (Figure 5A). Out of 920 proteins detected, 52 showed significant changes in expression levels, representing approximately 5.65% of the total proteome examined (Figure 5B). Consistent with the above observed effects of colchicine on C3H10T1/2 cells, differently expressed proteins were associated with adipogenesis, microtubule function, and autophagy, among other processes (Figure 5C–E) (Supplementary Tables S2–S5).

Focusing on microtubule-driven processes, during adipogenic differentiation and colchicine stimulation, DDA proteomic enrichment analysis revealed that processes related to the cell cycle were enriched (Supplementary Tables S2 and S3). Furthermore, pathways associated with microtubule structure were identified in SWATH proteomic enrichment analysis. Notably, colchicine downregulated pathways linked to microtubule stability (Supplementary Tables S4 and S5). In addition, colchicine altered the expression of proteins related to microtubules, including spectrin beta (SPTB1), proliferation-associated protein 2G4 (PA2G4), T-complex protein 1 subunit zeta (TCPZ), serine/threonine-protein phosphatase 2A (2ABA), filamin-C (FLNC), and myosin-9 (MYH9) (Figure 5C).

Regarding adipogenic differentiation, enrichment analysis identified the modulation of pathways closely related to adipogenesis (Supplementary Tables S2–S5). These results underscore the inhibitory impact of colchicine on adipogenesis, which is further supported by the downregulation of proteins associated with adipogenesis, such as alpha-2-macroglobulin receptor-associated protein (AMRP), hydroxymethylglutaryl-CoA synthase (HMCS1), ATP-citrate synthase (ACLY), very-long-chain (3R)-3-hydroxyacyl-CoA dehydratase 2 (HACD2), adipocyte plasma membrane-associated protein (APMAP), lanosterol 14-alpha demethylase (CP15A), integrin alpha-6 (ITA6), and NADP-dependent malic enzyme (MAOX) (Figure 5D).

Interestingly, enrichment analysis revealed that colchicine is strongly linked to autophagic processes (Supplementary Tables S2–S5). According to this, colchicine exposure induced the expression of autophagic proteins, including elongation factor Tu (EFTU), protein S100-A4 (S10A4), calcium load-activated calcium channel (TMCO1), ferritin heavy chain (FRIH), myosin-9 (MYH9), and clathrin heavy chain (CLH1) (Figure 5E).

## 4. Discussion

In this work, we have demonstrated that therapeutic concentrations of colchicine strongly inhibit adipogenesis while slightly promoting osteoblastogenesis. At these concentrations, colchicine altered microtubule structure and subcellular localization. Consistent with this, the dysregulation of microtubule homeostasis by STMN1 silencing also recapitulated the inhibition of adipogenesis. Interestingly, a transient stimulation with colchicine at the beginning of adipogenic differentiation was enough to completely abolish intracellular lipid accumulation and reduce the expression of several adipogenic marker genes. Moreover, colchicine exposure triggered changes in the expression of proteins related to adipogenesis, microtubules, and autophagy processes.

Gouty joints are characterised by bone erosions alongside increased bone anabolism, resulting in the formation of bone spurs and osteophytes [2,3,4,25,36]. This enhanced anabolism has been associated with the use of colchicine in these patients. In fact, in vivo studies have revealed that colchicine treatment can increase the expression of pro-osteoblastic markers and contribute to tissue mineralization [17,28,29]. Within the bone, osteoblastogenesis and adipogenesis are two antithetical processes that are mutually controlled [19]. However, despite colchicine’s known effects on osteoblast metabolism, little is known about its impact on adipocyte metabolism. Accordingly, we decided to explore the effect of colchicine on adipocyte metabolism and its potential use to control excessive bone adiposity, a well-known problem of multiple bone catabolic diseases and conditions.

Given that osteoblastogenesis and adipogenesis are mutually exclusive processes [19], we sought to gain further insights into the action of colchicine in bone by studying its effect on adipogenic differentiation. Several studies have suggested that colchicine may increase lipolytic activity, reduce plasma lipid levels [37,38], and promote fat browning [6]. However, the action of colchicine on the adipogenic process has not been explored to date. Interestingly, we observed that continuous colchicine stimulation of mesenchymal progenitors prevented the acquisition of an adipocytic phenotype, as evidenced by the loss of lipid droplet formation. Furthermore, in these cells, colchicine inhibited the expression of all the adipogenic marker genes studied.

Proteomic analysis confirmed the inhibitory effect of continuous colchicine stimulation on adipogenic differentiation. Several key adipocyte proteins were significantly inhibited after colchicine stimulation, including APMAP [39], ITA6 [40], and HACD2 [41], which is in accordance with the effect of colchicine on adipocyte size [42] and lipid droplet formation [43]. Colchicine also abolished the expression of SPTB1, a cytoskeleton protein that is closely related to lipid droplet formation [44]. Supporting these data, enrichment analysis showed that colchicine disrupts pathways involved in lipid metabolism, such as “lipid and atherosclerosis”, “fatty acid degradation”, and “peroxisome”. Routes like “plasma lipoprotein clearance” and “thermogenesis” were also enriched in our cellular model, which is in accordance with the lipolytic and thermogenic properties of colchicine described in the literature [6,37,45].

Importantly, these findings were also replicated using a single and transient stimulation of colchicine at the beginning of the adipogenic differentiation. The fact that a single, transient exposure to colchicine is sufficient to inhibit adipogenesis is particularly relevant, given the drug’s narrow therapeutic window and potential toxicity, and suggests that prolonged systemic dosing may not be necessary to elicit this cellular effect. While these results are limited to an in vitro model, they provide a rationale to explore short, site-specific interventions in preclinical settings where poor bone quality and high marrow adiposity can be locally targeted, such as during orthopaedic implantation procedures in compromised bone tissue. A brief colchicine exposure at the time of implantation could suppress adipogenesis in the surrounding microenvironment, potentially improving bone integration and reducing the risk of implant failure.

Regarding the mechanisms underlying this anti-adipogenic effect, the microtubule network plays a crucial role in maintaining cell shape, enabling cell division, and regulating intracellular trafficking, all of which are known to influence stem cell fate decisions [30]. Accordingly, cytoskeletal remodelling is essential for adipocyte development [46] and, consequently, microtubule disassembly has been demonstrated to impair adipogenesis [34]. In our cellular model, colchicine treatment changed the intracellular distribution of tubulin and actin. Specifically, tubulin changed its localization to the nucleus when cells were exposed to colchicine. Colchicine is known to cause microtubule depolymerization, which leads to the accumulation of tubulin in the nucleus [47] and blocks the cell cycle at the G2/M phase [9,10]. Accordingly, the expression of proteins related to microtubule organisation and cell division was diminished after colchicine exposure, confirming that colchicine disrupts microtubule stability [48]. In addition, pathway enrichment further supported this, with pathways such as “G2/M Checkpoints” and “chromosome organisation” being downregulated after colchicine treatment.

Adipogenic differentiation depends on precise regulation of cell-cycle dynamics, including mitotic clonal expansion and subsequent cell-cycle exit [49]. Thus, colchicine-induced proliferation inhibition may contribute to the anti-adipogenic effects observed. Nevertheless, proliferation control alone is unlikely to fully account for this phenotype, as cytoskeletal remodelling plays a central role in both cell-cycle regulation and cell differentiation commitment. Notably, inhibition of cell-cycle progression does not universally block adipogenesis; for example, CDK1 knockdown reduces proliferation while enhancing adipocyte formation in human mesenchymal stem cells [50]. These observations suggest that colchicine’s anti-adipogenic effect cannot be explained solely by growth arrest and may involve additional mechanisms related to cytoskeletal dynamics.

The same anti-adipogenic effect of colchicine was observed after disrupting microtubule homeostasis through STMN1 silencing, which also suppressed lipid droplet formation and reduced the expression of most adipogenic markers, suggesting that the anti-adipogenic effect of colchicine could be mediated by microtubule destabilisation. However, it should be noted that, although STMN1 silencing recapitulates key aspects of colchicine action on the microtubule cytoskeleton, it does not fully phenocopy its broader pharmacological effects. In this regard, and despite STMN1’s ability to regulate the microtubule assembly/disassembly balance [51], STMN1 silencing did not affect osteoblast commitment in our cellular model. In contrast with this observation, Liu et al. observed that global STMN1 knockout mice showed an osteopenic phenotype [29]. This discrepancy may be explained by differences in the degree, duration, and cell specificity of STMN1 inhibition in our in vitro model compared with the STMN1^−/−^ mouse model. Notably, STMN1 silencing in vitro captures the local, cell-autonomous effects of microtubule regulation in mesenchymal progenitors, whereas global STMN1 knockout models may involve additional systemic and neurogenic mechanisms that indirectly influence bone homeostasis. Nonetheless, it is worth noting that, in our cellular model, adipogenesis is more readily affected by colchicine and microtubule disruption than osteoblastogenesis.

Beyond the regulation of adipogenesis and the microtubule cytoskeleton, proteomic analysis revealed an increased expression of proteins positively associated with autophagy following colchicine stimulation. Additionally, pathway enrichment analysis indicated that several pathways related to autophagy, senescence, and mTOR signalling were also altered. However, in the absence of dedicated autophagy flux assays, it is not possible to determine whether these changes reflect increased autophagy activation or accumulation of autophagosomes due to impaired vesicular trafficking. Accordingly, we interpret these proteomic changes as evidence of autophagy pathway modulation, which is consistent with the known effects of colchicine on microtubule dynamics and vesicular transport. In line with this, colchicine has been demonstrated to modulate autophagy by increasing autophagy flux and blocking mTOR signalling [10]. Specifically, colchicine has been described to enhance the process of autophagosome formation and increase the formation of lysosomes [11]. In contrast, other authors have found that colchicine impairs autophagy by blocking the maturation of autophagosomes to autolysosomes, which provokes the accumulation of autophagic substrates [12]. Importantly, certain research findings indicate that selectively triggering or inhibiting autophagy can change cell fate. In the context of adipogenesis, autophagy regulates the transition from pre-adipocytes to mature adipocytes [52]. Therefore, colchicine modulation of autophagy pathways could contribute to its anti-adipogenic effect. 

## 5. Conclusions

In this work, we demonstrated that colchicine alters microtubule structure and osteoblast-adipocyte differentiation of mesenchymal stem cells. Specifically, we showed that clinical therapeutic colchicine concentrations significantly inhibited adipogenesis, even after a single and transient stimulation. Furthermore, we revealed that this anti-adipogenic effect involved microtubule homeostasis alteration, suggesting that controlling microtubule assembly could be a tool to regulate the osteoblast-adipocyte balance. Although these observations derive from an in vitro model and require confirmation in relevant preclinical systems, they support investigating colchicine-based strategies to counteract excessive bone adiposity in disorders such as osteoporosis.

## Data Availability

The data used to support the findings of this study are contained within the article. Proteomic data have been deposited in the PRIDE repository (ProteomeXchange Consortium) under the accession code PXD072951. Other raw data are available from the corresponding author upon request.

## Supplementary Materials

The following supporting information can be downloaded at https://www.mdpi.com/article/10.3390/pharmaceutics18010119/s1, Figure S1: HPRT expression. (**A**,**B**) HPRT expression during C3H10T1/2 differentiation and colchicine stimulation, measured by RT-qPCR. (**C**,**D**) HPRT expression during C3H10T1/2 differentiation and STMN1 silencing, measured by RT-qPCR. Supplementary Methods: Protein identification and quantification by LC–MS/MS–DDA and SWATH–MS analysis. Table S1: Primers used for RT-qPCR. Table S2: Pathway analysis of DDA proteomics results. Proteomic changes induced in C3H10T1/2 cells exposed to colchicine (25 nM) during a 7-day adipogenic differentiation, compared to other samples, analysed using KEGG and Reactome databases. Table S3: Pathway analysis of DDA proteomics results. Proteomic changes induced in C3H10T1/2 cells during a 7-day adipogenic differentiation compared to other samples, analysed using KEGG and Reactome databases. Table S4: Pathway analysis of SWATH–MS proteomics results. Proteomic changes induced in C3H10T1/2 cells exposed to colchicine (25 nM) during a 7-day adipogenic differentiation, compared to other samples, analysed using KEGG and Reactome databases. Table S5: Pathway analysis of SWATH–MS proteomics results. Proteomic changes induced in C3H10T1/2 cells during a 7-day adipogenic differentiation compared to other samples, analysed using KEGG and Reactome databases.

## Abbreviations

The following abbreviations are used in this manuscript:

BMP-2	Bone morphogenetic protein 2
DMEM-HG	Dulbecco’s Modified Eagle’s Medium High Glucose
DMEM F12	Dulbecco’s Modified Eagle’s Medium supplemented with Ham’s F-12
FBS	Foetal Bovine Serum
IGF-1	Insulin-like growth factor-1
IBMX	3-Isobutyl-1-methylxanthine
MTT	3-[4,5-Dimethylthiazol-2-yl]-2,5-Diphenyltetrazolium Bromide
OB	Osteoblastogenic
AD	Adipogenic
ADm	Adipogenic maintenance medium
PCR	Polymerase chain reaction
RT-qPCR	Real-time quantitative polymerase chain reaction
RNA	Ribonucleic acid
STMN1	Stathmin 1
SSP1	Osteopontin
RUNX2	Runt-related transcription factor 2
GPNMB	Glycoprotein non-metastatic melanoma B
LIF	Leukaemia inhibitory factor
PPARγ	Peroxisome proliferator-activated receptor gamma
PLIN2	Perilipin 2
ADIPOQ	Adiponectin
FABP4	Fatty acid-binding protein 4
SWATH-MS	Sequential Window Acquisition of All Theoretical Mass Spectra
DDA	Data-dependent acquisition
HPRT	Hypoxanthine phosphoribosyltransferase 1
SPTB1	Spectrin beta 1
PA2G4	Proliferation-associated protein 2G4
TCPZ	T-complex protein 1 subunit zeta
FLNC	Filamin-C
MYH9	Myosin-9
APMAP	Adipocyte plasma membrane-associated protein
ITA6	Integrin alpha-6
ACLY	ATP-citrate lyase
HACD2	(3R)-3-hydroxyacyl-CoA dehydratase 2
MAOX	NADP-dependent malic enzyme
EFTU	Elongation factor Tu
S100-A4	Protein S100-A4
TMCO1	Calcium load-activated calcium channel
FRIH	Ferritin heavy chain
CLH1	Clathrin heavy chain
