# Supplementary material for: Colchicine Suppresses Adipogenic Differentiation of Mesenchymal Stem Cells: Implications for Bone Adiposity Control
Source: Pharmaceutics. 2026 Jan 16;18(1):119. doi: 10.3390/pharmaceutics18010119 (PMC12844964; doi:10.3390/pharmaceutics18010119)
Supplement: Supplementary file 1 [file pharmaceutics-18-00119-s001.zip › pharmaceutics-4091094-SupplementaryMaterials.pdf]

Protein identification Quantification by LC-MS/MS-DDA analysis

Protein extraction

Protein extracts from the adipogenic differentiation experiments (including a day 0 control, a day 7 adipogenesis control, and a day 7 adipogenesis sample treated with 25 nM colchicine) were subjected to proteomic analysis. At the end of the treatment period, culture medium was removed and cells were gently washed with phosphate-buffered saline (PBS) to remove residual medium. Cells were lysed directly in the culture wells using RIPA buffer (Millipore, Sigma-Aldrich) supplemented with a protease and phosphatase inhibitor cocktail (Thermo Fisher Scientific). Lysis was initiated by incubating the cells with RIPA buffer for 5 min at room temperature, followed by mechanical disruption using a cell scraper to maximise protein recovery. The resulting lysates were then incubated on ice for 20 min to ensure complete cell lysis, with brief centrifugation steps performed every 5 min to facilitate disruption and reduce sample viscosity. Finally, cell debris was removed by centrifugation at 18,000 × g for 20 min at 4 °C. The clarified supernatants containing soluble proteins were carefully collected and used for downstream proteomic analyses.

Protein digestion

In order to make global protein identification and quantification, an equal amount of protein (over 100 μg), of 3 biological replicates from cells (D0_control, 7AD_control, and 7AD_colchicine 25mM) was loaded on a 10% SDS-PAGE gel. The run was stopped as soon as the front had penetrated 3 mm into the resolving gel. The protein band was detected by Sypro-Ruby fluorescent staining (Lonza, Switzerland), excised, and processed for in-gel, manual tryptic digestion, gel pieces were reduced with 10 mM dithiothreitol (Sigma-Aldrich, St. Louis, MO) in 50 mM ammonium bicarbonate (Sigma-Aldrich, St. Louis, MO) and alkylated with 55 mM iodoacetamide (Sigma- Aldrich, St. Louis, MO) in 50 mM ammonium bicarbonate. Then, the gel pieces were rinsed with 50 mM ammonium bicarbonate in 50% methanol (HPLC grade, Scharlau, Barcelona, Spain), dehydrated by addition of acetonitrile (HPLC grade, Scharlau, Barcelona, Spain) and dried in a SpeedVac. Modified porcine trypsin (Promega, Madison, WI, USA) was added to the dry gel pieces at a final concentration of 20 ng/μl in 20mM ammonium bicarbonate, incubating them at 37 °C for 16 h. Peptides were extracted thrice by 20 min incubation in 40 μL of 60% acetonitrile in 0.5% HCOOH. The resulting peptide extracts were pooled, concentrated in a SpeedVac and stored at −20 °C.

Protein quantification by SWATH (Sequential Window Acquisition of all Theoretical Mass Spectra)

Creation of the spectral library

In order to build the MS2 (MS/MS spectral libraries) spectral libraries, the peptide solutions were analyzed by a shotgun **data-dependent acquisition (DDA)** approach using **micro-LC-MS/MS**. To obtain a good representation of the peptides and proteins present in all samples, pooled vials of samples from each group was prepared using equal mixtures of the original samples. One 4 μL of each pool (D0_control, 7AD_control, and 7AD_colchicine 25mM) separated into a micro-LC system Ekspert nLC425 (Eksigen, Dublin, CA, USA) using an Eksigent C18 150 × 0.30 mm, 3 mm particle size and 120 Å pore size (Eksigent, Sciex) at a flow rate of 5μL/min. Water and ACN, both containing 0.1% formic acid, was used as solvents A and B, respectively. The gradient run consisted of 5% to 95% B for 30 min, 5 min at 90% B and finally 5 min at 5% B for column equilibration, for a total run time of 40 min. As the peptides eluted, they were directly injected into a hybrid quadrupole-TOF mass spectrometer Triple TOF 6600 (Sciex, Redwood City, CA, USA) operated with a data-dependent acquisition system in positive ion mode. A Micro source (Sciex) was used for the interface between microLC and MS, with an application of 2600 V voltage. The acquisition mode consisted of a 250 ms survey (MS scan) MS1 scan from 400 to 1250 m/z followed by an (MSMS Scan) MS2 scan from 100 to 1500 m/z (25 ms acquisition time) of the top 65 precursor ions from the survey scan, for a total cycle time of 2.8 s. The fragmented precursors were then added to a dynamic exclusion list for 15 s; any singly charged ions were excluded from the (MS/MS analysis) MS2 analysis.

The peptide and protein identifications was performed using Protein Pilot software (version 5.0.1, Sciex) with a Data was searched using a Mouse specific Uniprot database, specifying iodoacetamide as Cys alkylation as variable modification and metionin oxidation as fixed modification. False discovery rate was performed using a nonlinear fitting method displaying only those results that reported a 1% global false discovery rate or better. The MS2 spectra (MS/MS spectra) of the identified peptides were then used to generate the spectral library for SWATH peak extraction using the add-in for PeakView Software (version 2.2, Sciex) MS/MS^{ALL} with SWATH Acquisition MicroApp (version 2.0, Sciex). Only peptides with a confidence score above 99% (as obtained from Protein Pilot database search FDR<1%) were included in the spectral library. This library was created to obtain the fragment ion intensity and retention time dimensions essentially to make the SWATH method.

Relative quantification by SWATH acquisition

SWATH (Sequential Window Acquisition of all Theoretical Mass Spectra) – MS acquisition was performed on a TripleTOF® 6600 LC-MS/MS system (Sciex). 4 µL of Peptides from each individual RBCs samples were analyzed using a **data-independent acquisition (IDA)**. Each sample (4 µL) was analyzed using the LC-MS equipment and LC gradient described above for building the spectral library but instead using the SWATH-MS acquisition method. The method consisted of repeating a cycle that consisted of the acquisition of 65 TOF (MS/MS scans) MS2 scans (400 to 1500 m/z, high sensitivity mode, 50 ms acquisition time) of overlapping sequential precursor isolation windows of variable width (1 m/z overlap) covering the 400 to 1250 m/z mass range with a previous TOF MS1 scan (400 to 1500 m/z, 50 ms acquisition time) for each cycle. Total cycle time was 6.3 s. For each sample set, the width of the **100 variable windows** was optimized according to the ion density found in the DDA runs using a SWATH variable window calculator worksheet from Sciex. SWATH quantification was attempted for all proteins in the ion library that were identified by ProteinPilot with an FDR below 1%.

Data analysis

The targeted data extraction of the fragment ion chromatogram traces from the SWATH runs was performed by PeakView (version 2.2, Sciex) using the SWATH Acquisition MicroApp (version 2.0). This application processed the data using the spectral libaray. PeakView computed an FDR and a score for each assigned peptide according to the chromatographic and spectra components; only peptides with an FDR below 1% were used for protein quantization; any shared and modified peptides were excluded from the processing. The retention times from the peptides that were selected for each protein was realigned in each run according to the iRT peptides corresponding to different identified proteins in each sample and eluted along the whole-time axis. Five-minute windows and 30 ppm widths were used to extract the ion chromatograms. Them extracted ion chromatograms were generated for each selected fragment ion; the peak areas for the protein were obtained by summing the peak areas **from 10 peptides (MS1 scan) and 7 corresponding fragment ions/transitions (MS2 scan) from each peptide**. Them this integrated peak areas (processed. mrkvw files from PeakView) were directly exported to the MarkerView 1.3.1 software (Sciex, CA, USA) for relative quantitative analysis. The export data will generate three files containing quantitative information about individual ions, the summed intensity of different ions for a particular peptide and the summed intensity of different peptides for a particular protein. The integrated peak areas (processed. mrkvw files

from PeakView) were directly exported to the MarkerView software (AB SCIEX) for relative quantitative analysis. The export will generate three files containing quantitative information about individual ions, the summed intensity of different ions for a particular peptide and the summed intensity of different peptides for a particular protein. MarkerView has been used for analysis of SWATH-MS data because of its data-independent method of quantitation. MarkerView uses processing algorithms that accurately find chromatographic and spectral peaks direct from the raw SWATH data. Data alignment by MarkerView compensates for minor variations in both mass and retention time values, ensuring that identical compounds in different samples are accurately compared to one another. Unsupervised multivariate statistical analysis using principal component analysis was performed to compare the data from the different samples. A **most-like ratio normalization** was performed after statistical analysis to control for possible uneven sample loss across the different samples during the sample preparation process. The average MS peak area for each protein was derived from each sample, followed by analysis using a Student's t-test (MarkerView 1.3.1 software, Sciex, Redwood City, CA, USA) to compare between samples based on the averaged total area of all transitions for each protein.

The t-test result (p-value) indicates the differences between the two groups (D0_Control vs. 7AD_Control or 7AD_Control vs. 7AD_Colchicine). A significance threshold of $p < 0.05$ (-log10(p-value)) and cut-off a symmetric fold-change threshold of $|\log2FC| \geq 0.5$ were applied.

Protein Functional Enrichment Analysis

Functional enrichment analyses were performed using two complementary proteomic workflows. For the DDA dataset, proteins exclusively identified in each experimental group (7AD_Control and 7AD_Colchicine) were selected and subjected to pathway enrichment analysis using the Kyoto Encyclopedia of Genes and Genomes (KEGG) and Reactome databases. These analyses were conducted in RStudio, and pathways were considered significantly enriched at $p < 0.05$.

For the SWATH dataset, functional enrichment analyses using KEGG and Reactome databases were also performed in RStudio. Enrichment was carried out by comparing proteins exclusively detected in the 7AD_Control versus all other groups, as well as proteins exclusively detected in the 7AD_Colchicine condition versus all other groups. This approach enabled the identification of biological processes and pathways significantly altered between conditions ($p < 0.05$).

Supplementary Figure 1

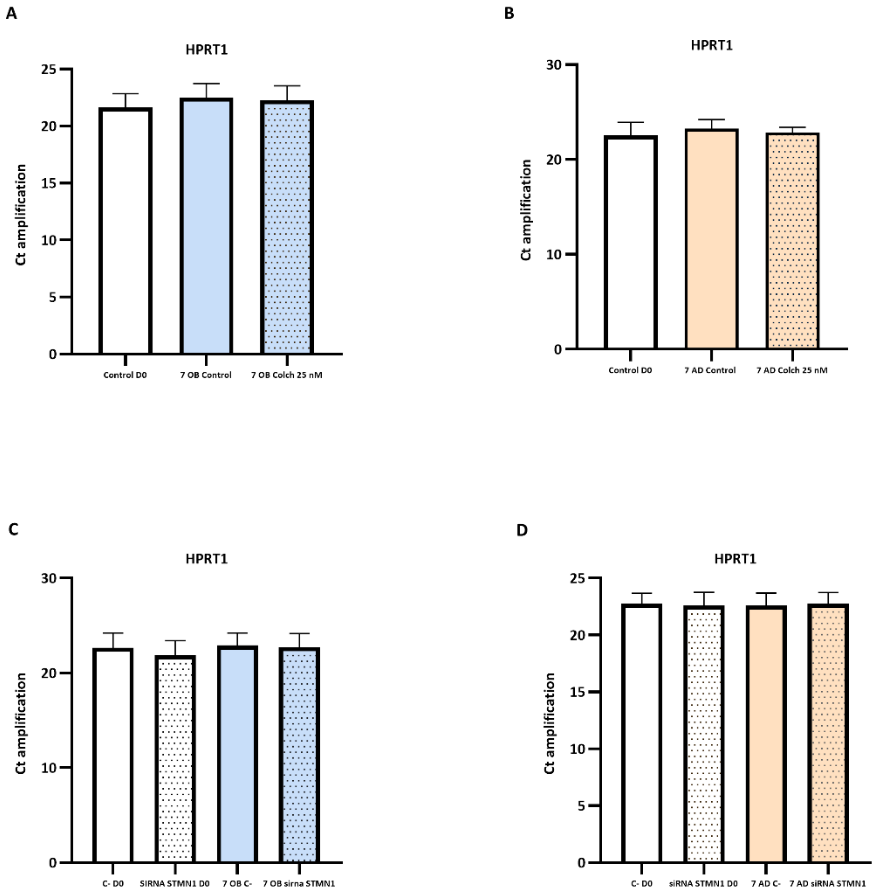

Figure S1: HPRT expression. (A–B) HPRT expression during C3H10T1/2 differentiation and colchicine stimulation, measured by RT-qPCR. (C–D) HPRT expression during C3H10T1/2 differentiation and STMN1 silencing, measured by RT-qPCR.

Table S1: Primers used for RT-qPCR.

Description	Symbol	Forward Primer (5′-3′) Sequence	Reverse Primer (5′-3′) Sequence
**Mouse Osteopontin**	SPP1	GGATGAATCTGACGAATCTC	GCATCAGGATACTGTTCATC
**Mouse Osteoactivin**	GPNMB	CTCTTTAATGCCTACTGGTTAC	GCCATATCTGTTTATTCGGC
**Mouse RUNT-related transcription factor 2**	RUNX2	ACAAGGACAGAGTCAGATTAC	CAGTGTCATCATCTGAAATACG
**Mouse Fatty acid binding protein 4**	FABP4	GTAAATGGGGATTTGGTCAC	TATGATGCTCTTCACCTTCC
**Mouse Perilipin 2**	PLIN2	ATAAGCTCTATGTCTCGTGG	GCCTGATCTTGAATGTTCTG
**Mouse Adiponectin**	ADIPOQ	CCACTTTCTCCTCATTTCTG	CTAGCTCTTCAGTTGTAGTAAC
**Mouse Peroxisome proliferator activator receptor γ**	PPARG	AAAGACAACGGACAAATCAC	GGGATATTTTTGGCATACTCTG
**Mouse stahtmin 1**	STMN1	GATATTCAGGTGAAAGAGCTG	GAAAGGTCCTTCTTCTTTGG
**Mouse Hypoxanthine Phosphoribosyl transferase 1**	HPRT1	AGGGATTTGAATCACGTTTG	TTTACTGGCAACATCAACAG

Table S2: Pathway analysis of DDA proteomics results. Proteomic changes induced in C3H10T1/2 cells exposed to colchicine (25 nM) during a 7-day adipogenic differentiation compared to other samples, analysed using KEGG and Reactome databases.

	Description	p-value	Count
KEGG database	Protein processing in endoplasmic reticulum	0.00011859	8
	Mitophagy - animal	0.00362235	4
	Peroxisome	0.00521628	4
	Valine, leucine and isoleucine degradation	0.0099702	3
	Steroid biosynthesis	0.01284603	2
	Butanoate metabolism	0.01466619	2
	Terpenoid backbone biosynthesis	0.01861667	2
	Glycerolipid metabolism	0.01923541	3
	Bacterial invasion of epithelial cells	0.02330325	3
	Endocytosis	0.02695306	6
	RNA degradation	0.02780297	3
	Glycerophospholipid metabolism	0.04824736	3
Reactome database	Cholesterol biosynthesis	0.0001516	4
	Cargo recognition for clathrin-mediated endocytosis	0.00037489	6
	Clathrin-mediated endocytosis	0.00196213	6
	Nuclear Envelope (NE) Reassembly	0.00359295	4
	DNA Damage Recognition in GG-NER	0.00446572	3
	Receptor Mediated Mitophagy	0.00500821	2
	Glycerophospholipid biosynthesis	0.0054848	5
	Macroautophagy	0.00678565	5
	Autophagy	0.00678565	5
	N-glycan trimming in the ER and Calnexin/Calreticulin cycle	0.00931934	2
	Deadenylation-dependent mRNA decay	0.01607434	3
	MET activates PTK2 signaling	0.01633395	2
	Postmitotic nuclear pore complex (NPC) reformation	0.01793872	2
	Acyl chain remodelling of PS	0.01960744	2
	Collagen biosynthesis and modifying enzymes	0.02126229	3
	Glycolysis	0.02126229	3
	Regulation of HSF1-mediated heat shock response	0.02220597	3
	L1CAM interactions	0.02416121	3
	Selective autophagy	0.02620693	3
	Sealing of the nuclear envelope (NE) by ESCRT-III	0.02689429	2
	Mitophagy	0.03296483	2
	Regulation of Glucokinase by Glucokinase Regulatory Protein	0.03509682	2
	MET promotes cell motility	0.03509682	2
	Endosomal Sorting Complex Required For Transport	0.03509682	2

	(ESCRT)		
	Collagen formation	0.03529021	3
	Axon guidance	0.03623711	6
	Global Genome Nucleotide Excision Repair (GG-NER)	0.03652614	3
	Metabolism of steroids	0.03669307	4
	Nervous system development	0.03684355	6
	Nuclear Pore Complex (NPC) Disassembly	0.03728097	2
	Phospholipid metabolism	0.03806714	5
	Transcriptional regulation by small RNAs	0.03951607	2
	Transport of the SLBP independent Mature mRNA	0.04180094	2
	SUMOylation of SUMOylation proteins	0.04180094	2
	Lysosome Vesicle Biogenesis	0.04180094	2
	Cellular response to heat stress	0.04303594	3
	Glucose metabolism	0.04303594	3
	Transport of the SLBP Dependant Mature mRNA	0.04413439	2
	Recycling pathway of L1	0.04651527	2
	Collagen chain trimerization	0.04651527	2

Table S3: Pathway analysis of DDA proteomics results. Proteomic changes induced in C3H10T1/2 cells during a 7-day adipogenic differentiation compared to other samples, analysed using KEGG and Reactome databases.

	Description	p-value	Count
KEGG database	Diabetic cardiomyopathy	1.4452E-10	18
	Lipoic acid metabolism A2:C34	3.8923E-10	23
	Parkinson disease	7.8763E-10	19
	Prion disease	6.521E-09	18
	Amyotrophic lateral sclerosis	6.5721E-09	21
	Chemical carcinogenesis - reactive oxygen species	1.9044E-08	16
	Huntington disease	4.4395E-08	18
	Non-alcoholic fatty liver disease	8.2486E-08	13
	Pathways of neurodegeneration	9.7738E-08	22
	Oxidative phosphorylation	1.3159E-07	12
	Thermogenesis	2.168E-07	15
	Retrograde endocannabinoid signaling	0.00010991	9
	Nucleotide metabolism	0.00068786	6
	Autophagy - other	0.00068787	4
	Autophagy - animal	0.00222064	7
	Small cell lung cancer	0.00662273	5
	Purine metabolism	0.00726425	6
	Lysosome	0.00752474	6
	SNARE interactions in vesicular transport	0.00836581	3
	Spinocerebellar ataxia	0.00923151	6
	AGE-RAGE signaling pathway in diabetic complications	0.00931598	5
	Pentose and glucuronate interconversions	0.01064953	3
	Nucleocytoplasmic transport	0.01679594	5
	One carbon pool by folate	0.02396822	2
	Lipoic acid metabolism	0.02396822	2
	Amino sugar and nucleotide sugar metabolism	0.02707337	3
	Drug metabolism - other enzymes	0.02781959	4
	Longevity regulating pathway	0.02781959	4
	Cellular senescence	0.03020121	6
	Sphingolipid metabolism	0.031377	3
	Taurine and hypotaurine metabolism	0.03158652	2
	mRNA surveillance pathway	0.04250343	4
	Biosynthesis of cofactors	0.04483642	5
	Amoebiasis	0.04775407	4
Reactome database	Respiratory electron transport	1.0941E-08	12
	Respiratory electron transport, ATP synthesis by chemiosmotic coupling, and heat production by uncoupling proteins.	1.4872E-08	13
	The citric acid (TCA) cycle and respiratory electron transport	1.2334E-07	14
	Complex I biogenesis	1.3441E-06	8
	Nucleotide catabolism	0.00173808	4

	Purine catabolism	0.00225508	3
	TP53 Regulates Metabolic Genes	0.00407503	5
	Apoptotic execution phase	0.00556449	4
	Energy dependent regulation of mTOR by LKB1-AMPK	0.00733047	3
	Mitochondrial translation elongation	0.0078881	5
	Mitochondrial translation termination	0.0087043	5
	Mitochondrial translation	0.00913355	5
	Macroautophagy	0.00938361	6
	Autophagy	0.00938361	6
	SUMOylation of immune response proteins	0.01115099	2
	Molecules associated with elastic fibres	0.01177637	3
	Metabolism of nucleotides	0.01310505	5
	Asparagine N-linked glycosylation	0.01431626	9
	Apoptotic cleavage of cellular proteins	0.01623709	3
	ER to Golgi Anterograde Transport	0.02003798	6
	Elastic fibre formation	0.02012355	3
	MTOR signalling	0.02152294	3
	Metabolism of Angiotensinogen to Angiotensins	0.02317453	2
	Biosynthesis of DHA-derived SPMs	0.02601115	2
	Nicotinamide salvaging	0.02898066	2
	Branched-chain amino acid catabolism	0.02898066	2
	Biosynthesis of specialized proresolving mediators (SPMs)	0.02898066	2
	Processing of Intronless Pre-mRNAs	0.03530028	2
	Role of phospholipids in phagocytosis	0.03864164	2
	Synaptic adhesion-like molecules	0.03864164	2
	Postmitotic nuclear pore complex (NPC) reformation	0.03864164	2
	Ubiquitin-dependent degradation of Cyclin D	0.04033558	3
	Transport to the Golgi and subsequent modification	0.0427513	6
	Programmed Cell Death	0.04370817	5
	CLEC7A (Dectin-1) signaling	0.04543068	4
	Translation	0.04646088	7
	COPI-mediated anterograde transport	0.04699069	4
	VEGFA-VEGFR2 Pathway	0.04858029	4
	mTORC1-mediated signalling	0.04934136	2

Table S4: Pathway analysis of SWATH–MS proteomics results. Proteomic changes induced in C3H10T1/2 cells exposed to colchicine (25 nM) during a 7-day adipogenic differentiation compared to other samples, analysed using KEGG and Reactome databases.

	Description	p-value
KEGG database	Carbon metabolism	5.4421E-10
	Biosynthesis of amino acids	6.2758E-08
	Valine, leucine and isoleucine degradation	1.1466E-07
	Glycolysis / Gluconeogenesis	3.5711E-07
	HIF-1 signaling pathway	1.3074E-05
	PPAR signaling pathway	3.335E-05
	Arginine and proline metabolism	3.4051E-05
	Citrate cycle (TCA cycle)	6.6527E-05
	2-Oxocarboxylic acid metabolism	8.4905E-05
	Pyruvate metabolism	0.00023589
	Fatty acid degradation	0.00045122
	Terpenoid backbone biosynthesis	0.00051677
	Butanoate metabolism	0.00083671
	Fatty acid metabolism	0.0008826
	Propanoate metabolism	0.00126054
	Pentose phosphate pathway - Mus musculus (house mouse)	0.00151493
	Peroxisome	0.00309304
	Proteasome	0.00420094
	Alzheimer disease	0.00486881
	Protein processing in endoplasmic reticulum	0.00662878
	Pantothenate and CoA biosynthesis	0.00920802
	Parkinson disease	0.00989956
	Lysine degradation	0.00992679
	Prion disease	0.01043125
	Renal cell carcinoma	0.01170817
	Central carbon metabolism in cancer	0.01218041
	Focal adhesion	0.01331867
	Ascorbate and aldarate metabolism	0.01950013
	Galactose metabolism	0.02070786
	beta-Alanine metabolism	0.02070786
	Starch and sucrose metabolism	0.02321442
	Biosynthesis of unsaturated fatty acids	0.02321442
	ECM-receptor interaction	0.02393897
	Biosynthesis of nucleotide sugars	0.02857885
	Pathways of neurodegeneration	0.04450814
	Cholesterol metabolism	0.04722865
	Amino sugar and nucleotide sugar metabolism	0.04894044

Reactome database	Metabolism of amino acids and derivatives	1.6798E-06
	Glycolysis	7.5947E-05
	Gluconeogenesis	0.00010752
	Glucose metabolism	0.00028135
	Branched-chain amino acid catabolism	0.0002924
	Collagen biosynthesis and modifying enzymes	0.00097902
	The citric acid (TCA) cycle and respiratory electron transport	0.00124844
	Interferon Signaling	0.00132226
	Oxygen-dependent proline hydroxylation of Hypoxia-inducible Factor Alpha	0.00139981
	Antiviral mechanism by IFN-stimulated genes	0.00150385
	NCAM signaling for neurite out-growth	0.00150385
	Cellular response to hypoxia	0.00174196
	Fatty acyl-CoA biosynthesis	0.00180622
	Collagen formation	0.00203391
	Laminin interactions	0.00234111
	Degradation of beta-catenin by the destruction complex	0.00247367
	Ketone body metabolism	0.00284772
	Regulation of mRNA stability by proteins that bind AU-rich elements	0.00354163
	Glutamate and glutamine metabolism	0.00400022
	Pyruvate metabolism and Citric Acid (TCA) cycle	0.00498715
	Cross-presentation of soluble exogenous antigens (endosomes)	0.00560743
	Cellular response to chemical stress	0.0058285
	NCAM1 interactions	0.00606681
	Regulation of ornithine decarboxylase (ODC)	0.00627209
	Ubiquitin-dependent degradation of Cyclin D	0.00627209
	GSK3B and BTRC:CUL1-mediated-degradation of NFE2L2	0.00627209
	Autodegradation of the E3 ubiquitin ligase COP1	0.00662132
	Ubiquitin Mediated Degradation of Phosphorylated Cdc25A	0.00662132
	p53-Independent DNA Damage Response	0.00662132
	p53-Independent G1/S DNA damage checkpoint	0.00662132
	Regulation of RUNX2 expression and activity	0.00662132
	Nuclear events mediated by NFE2L2	0.00662132
	Regulation of RUNX3 expression and activity	0.00698193
	Extracellular matrix organization	0.00704283
	FBXL7 down-regulates AURKA during mitotic entry and in early mitosis	0.00735402
	Degradation of AXIN	0.00773768
	Hedgehog 'off' state	0.00774253
	AUF1 (hnRNP D0) binds and destabilizes mRNA	0.00813299
	Degradation of GLI1 by the proteasome	0.00813299
	Stabilization of p53	0.00813299
	Cytokine Signaling in Immune system	0.00834448
	Degradation of DVL	0.00854003
	Dectin-1 mediated noncanonical NF-kB signaling	0.00854003
	GLI3 is processed to GLI3R by the proteasome	0.00854003

	NIK-->noncanonical NF-kB signaling	0.00854003
	G2/M Transition	0.00937054
	Metabolism of polyamines	0.00938959
	Mitotic G2-G2/M phases	0.00981394
	Interleukin-1 family signaling	0.0102566
	SCF(Skp2)-mediated degradation of p27/p21	0.01028692
	Citric acid cycle (TCA cycle)	0.0103674
	Asymmetric localization of PCP proteins	0.01075366
	Fc epsilon receptor (FCERI) signaling	0.01119949
	Activation of NF-kappaB in B cells	0.01123253
	Hedgehog ligand biogenesis	0.01123253
	Transcriptional regulation by RUNX2	0.01123253
	Synthesis of very long-chain fatty acyl-CoAs	0.01135011
	Autodegradation of Cdh1 by Cdh1:APC/C	0.01172359
	Metabolism of carbohydrates	0.01222314
	p53-Dependent G1 DNA Damage Response	0.01222688
	p53-Dependent G1/S DNA damage checkpoint	0.01222688
	RUNX1 regulates transcription of genes involved in differentiation of HSCs	0.01274245
	Regulation of RAS by GAPs	0.01327036
	G1/S DNA Damage Checkpoints	0.01327036
	Beta-catenin independent WNT signaling	0.01361288
	APC/C:Cdc20 mediated degradation of Securin	0.01381063
	Regulation of PTEN stability and activity	0.01381063
	Mitotic G1 phase and G1/S transition	0.01435868
	Orc1 removal from chromatin	0.01492846
	Cellular responses to stress	0.01515826
	Cyclin E associated events during G1/S transition	0.01550607
	Cellular responses to stimuli	0.01573799
	Cdc20:Phospho-APC/C mediated degradation of Cyclin A	0.01669887
	MAPK6/MAPK4 signaling	0.01669887
	CDK-mediated phosphorylation and removal of Cdc6	0.01669887
	Cyclin A:Cdk2-associated events at S phase entry	0.01669887
	ISG15 antiviral mechanism	0.01684224
	APC/C:Cdh1 mediated degradation of Cdc20 and other APC/C:Cdh1 targeted proteins in late mitosis/early G1	0.01731411
	APC:Cdc20 mediated degradation of cell cycle proteins prior to satisfation of the cell cycle checkpoint	0.01731411
	Downstream signaling events of B Cell Receptor (BCR)	0.01794193
	The role of GTSE1 in G2/M progression after G2 checkpoint	0.01794193
	KEAP1-NFE2L2 pathway	0.01794193
	Cholesterol biosynthesis	0.01805214
	APC/C:Cdc20 mediated degradation of mitotic proteins	0.01858236
	Transcriptional regulation by RUNX3	0.01858236
	Signaling by Hedgehog	0.01892415
	Activation of APC/C and APC/C:Cdc20 mediated degradation of mitotic	0.01923542

	proteins	
	FCERI mediated NF-kB activation	0.01923542
	Hedgehog 'on' state	0.02197414
	Assembly of the pre-replicative complex	0.02269049
	Neddylation	0.02313474
	Non-integrin membrane-ECM interactions	0.02324378
	TCF dependent signaling in response to WNT	0.02430261
	Downstream TCR signaling	0.02491559
	Signaling by WNT	0.02605596
	APC/C-mediated degradation of cell cycle proteins	0.02646234
	PCP/CE pathway	0.02646234
	Regulation of mitotic cell cycle	0.02646234
	CLEC7A (Dectin-1) signaling	0.02887735
	Activation of NMDA receptors and postsynaptic events	0.02897154
	Switching of origins to a post-replicative state	0.02970761
	Antigen processing-Cross presentation	0.03055047
	Respiratory electron transport	0.03140591
	UCH proteinases	0.03404752
	TNFR2 non-canonical NF-kB pathway	0.03495307
	DNA Replication Pre-Initiation	0.03495307
	ABC-family proteins mediated transport	0.03680153
	Signaling by the B Cell Receptor (BCR)	0.03774438
	TCR signaling	0.03966716
	Interleukin-1 signaling	0.03966716
	Intracellular signaling by second messengers	0.04006852
	G1/S Transition	0.04163914
	Assembly of collagen fibrils and other multimeric structures	0.04364113
	RAF/MAP kinase cascade	0.0441733
	Degradation of the extracellular matrix	0.04468869
	Ub-specific processing proteases	0.04481511
	C-type lectin receptors (CLRs)	0.04572947
	Signaling by Interleukins	0.0466293
	MAPK1/MAPK3 signaling	0.04788755
	PTEN Regulation	0.04892396

Table S5: Pathway analysis of SWATH–MS proteomics results. Proteomic changes induced in C3H10T1/2 cells during a 7-day adipogenic differentiation compared to other samples, analysed using KEGG and Reactome databases.

	Description	p-value
KEGG database	Parkinson disease	1.4424E-05
	Prion disease	9.9507E-05
	Chemical carcinogenesis - reactive oxygen species	0.00015813
	Huntington disease	0.00025214
	Drug metabolism	0.00041914
	Biosynthesis of cofactors	0.00068635
	Ribosome	0.00151294
	Oxidative phosphorylation	0.002591
	Glycine, serine and threonine metabolism	0.00276781
	Nucleotide metabolism	0.00287926
	Peroxisome	0.00326948
	Vasopressin-regulated water reabsorption	0.00363638
	Non-alcoholic fatty liver disease	0.00481487
	Pyrimidine metabolism	0.00716662
	Necroptosis	0.00777599
	Coronavirus disease - COVID-19	0.00810168
	Longevity regulating pathway	0.0090749
	Carbon metabolism	0.01043846
	Metabolism of xenobiotics by cytochrome P450	0.01477135
	Lysosome	0.01511191
	Butanoate metabolism	0.01542481
	Pathways of neurodegeneration	0.01739231
	Lipid and atherosclerosis	0.01807055
	Propanoate metabolism	0.02006833
	beta-Alanine metabolism	0.02130978
	Antigen processing and presentation	0.02564151
	Small cell lung cancer	0.02790536
	Salmonella infection	0.03291398
	Amoebiasis	0.0398309
	Toxoplasmosis	0.0417153
	Endocytosis	0.04228045
	Base excision repair	0.04341418
	Amyotrophic lateral sclerosis	0.04554189
	**Fatty acid degradation**	**0.05207603**
**Reactome database**	Association of TriC/CCT with target proteins during biosynthesis	8.5285E-11
	Detoxification of Reactive Oxygen Species	3.8219E-07
	Cooperation of PDCL (PhLP1) and TRiC/CCT in G-protein beta folding	6.58E-07
	Chaperonin-mediated protein folding	7.7981E-07

	Protein folding	7.7981E-07
	L13a-mediated translational silencing of Ceruloplasmin expression	4.4928E-06
	GTP hydrolysis and joining of the 60S ribosomal subunit	4.8177E-06
	Eukaryotic Translation Initiation	7.6997E-06
	Cap-dependent Translation Initiation	7.6997E-06
	Nonsense Mediated Decay (NMD) independent of the Exon Junction Complex (EJC)	1.4234E-05
	Formation of a pool of free 40S subunits	2.3283E-05
	Nonsense-Mediated Decay (NMD)	5.2576E-05
	Nonsense Mediated Decay (NMD) enhanced by the Exon Junction Complex (EJC)	5.2576E-05
	Interconversion of nucleotide di- and triphosphates	8.8579E-05
	SRP-dependent cotranslational protein targeting to membrane	0.00012677
	Metabolism of nucleotides	0.00018258
	Apoptosis	0.0002705
	TP53 Regulates Metabolic Genes	0.00041108
	Cellular response to chemical stress	0.00046355
	Translation	0.00073546
	RHO GTPases activate PKNs	0.00081343
	Programmed Cell Death	0.00099681
	Azathioprine ADME	0.00153995
	Cellular responses to stress	0.00168166
	Mitochondrial biogenesis	0.00172137
	Translation initiation complex formation	0.00173704
	Ribosomal scanning and start codon recognition	0.00173704
	Cellular responses to stimuli	0.00177541
	Activation of the mRNA upon binding of the cap-binding complex and eIFs, and subsequent binding to 43S	0.00185088
	RHO GTPase cycle	0.00242695
	RHOG GTPase cycle	0.00311752
	Transcriptional activation of mitochondrial biogenesis	0.00338858
	Respiratory electron transport, ATP synthesis by chemiosmotic coupling, and heat production by uncoupling proteins.	0.00370955
	Major pathway of rRNA processing in the nucleolus and cytosol	0.00426655
	rRNA processing	0.00426655
	rRNA processing in the nucleus and cytosol	0.00426655
	Intrinsic Pathway for Apoptosis	0.00462722
	Activation of BAD and translocation to mitochondria	0.00491271
	Chk1/Chk2(Cds1) mediated inactivation of Cyclin B:Cdk1 complex	0.00491271
	Ribavirin ADME	0.00491271
	Rap1 signalling	0.00669571
	Regulation of mRNA stability by proteins that bind AU-rich elements	0.00691652
	Apoptotic execution phase	0.00890129
	Respiratory electron transport	0.00945454
	LDL clearance	0.00983497

	HSP90 chaperone cycle for steroid hormone receptors (SHR) in the presence of ligand	0.010515
	Formation of the ternary complex, and subsequently, the 43S complex	0.010515
	Activation of BH3-only proteins	0.01100075
	Golgi Associated Vesicle Biogenesis	0.01108812
	Complex I biogenesis	0.01291448
	Pyroptosis	0.01350462
	RHOBTB2 GTPase cycle	0.01350462
	AUF1 (hnRNP D0) binds and destabilizes mRNA	0.01355922
	G2/M Checkpoints	0.01367858
	The citric acid (TCA) cycle and respiratory electron transport	0.01509327
	mTORC1-mediated signalling	0.0191714
	Selective autophagy	0.02018283
	Platelet activation, signaling and aggregation	0.02159413
	trans-Golgi Network Vesicle Budding	0.02272209
	ISG15 antiviral mechanism	0.02396523
	Antiviral mechanism by IFN-stimulated genes	0.03103064
	RHOBTB GTPase Cycle	0.03103064
	DDX58/IFIH1-mediated induction of interferon-alpha/beta	0.03291088
	Plasma lipoprotein clearance	0.03483482
	RHOV GTPase cycle	0.03483482
	Cell Cycle Checkpoints	0.03759572
	TAK1-dependent IKK and NF-kappa-B activation	0.03881016
	RHOU GTPase cycle	0.03881016
	Organelle biogenesis and maintenance	0.04081098
	G2/M DNA damage checkpoint	0.0426463
	Aggrephagy	0.04294952
	Signaling by NOTCH	0.04945099
	MTOR signalling	0.04945099
